# The mRNA Distribution of Cancer Stem Cell Marker CD90/Thy-1 Is Comparable in Hepatocellular Carcinoma of Eastern and Western Populations

**DOI:** 10.3390/cells9122672

**Published:** 2020-12-12

**Authors:** An B. Luong, Huy Q. Do, Paola Tarchi, Deborah Bonazza, Cristina Bottin, Loraine Kay D. Cabral, Long D. C. Tran, Thao P. T. Doan, Lory S. Crocè, Hoa L. T. Pham, Claudio Tiribelli, Caecilia H. C. Sukowati

**Affiliations:** 1 Fondazione Italiana Fegato ONLUS, AREA Science Park Basovizza, 34149 Trieste, Italy; luongbacan1991@gmail.com (A.B.L.); huydo0203@gmail.com (H.Q.D.); kay.cabral@fegato.it (L.K.D.C.); lcroce@units.it (L.S.C.); ctliver@fegato.it (C.T.); 2 Center for Molecular Biomedicine, University of Medicine and Pharmacy at Ho Chi Minh, Ho Chi Minh City 700000, Vietnam; 3 Laboratory of Stem Cell Research and Application, VNUHCM-University of Science, Ho Chi Minh City 700000, Vietnam; 4 Clinical Surgery Unit, Azienda Sanitaria Universitaria Giuliana Isontina (ASUGI), 34148 Trieste, Italy; paolatarchi@hotmail.com; 5 Surgical Pathology Unit, Azienda Sanitaria Universitaria Giuliana Isontina (ASUGI), 34148 Trieste, Italy; deborah.bonazza@asuits.sanita.fvg.it; 6 Department of Medical, Surgical and Health Sciences, University of Trieste, 34127 Trieste, Italy; cbottin@units.it; 7 Doctoral School in Molecular Biomedicine, University of Trieste, 34127 Trieste, Italy; 8 University Medical Center, University of Medicine and Pharmacy at Ho Chi Minh, Ho Chi Minh City 700000, Vietnam; long.tcd@umc.edu.vn (L.D.C.T.); hoaph59@gmail.com (H.L.T.P.); 9 Department of Pathology, University of Medicine and Pharmacy at Ho Chi Minh, Ho Chi Minh City 700000, Vietnam; thaodoanthiphuong@ump.edu.vn

**Keywords:** hepatocellular carcinoma, cancer stem cells, mRNA expression

## Abstract

Epidemiology of hepatocellular carcinoma (HCC) showed a correlation between incidence and geographical-relevant risk factors. This study aims to compare the distributions of cancer stem cells (CSC) in two distant populations in Asia and Europe. We analyzed 52 and 43 selected HCC patients undergoing hepatectomy in Ho Chi Minh City (Vietnam) and Trieste (Italy). Each patient sample consisted of HCC, peri-HCC, and non-tumoral (distal) tissue. Demographic data were recorded together with clinical findings. The protocol for the collection of tissue samples and RNA was standardized in both laboratories and gene expression analysis was performed in a single laboratory with identical PCR conditions. Baseline data showed comparable laboratory findings between the two cohorts. mRNA distribution showed a comparable pattern of all CSC markers analyzed with the expression of CD90 progressively increasing from distal and peri-HCC to be highest in HCC (*p* < 0.001), confirmed by immunofluorescence data. CD90 mRNA distribution was related to HBV-related HCC and a tumor diameter less than 5 cm. Patients with high tumoral CD90 mRNA had a shorter time (*p* < 0.05) to tumor recurrence compared to patients with lower CD90. This comparative study showed that CD90 mRNA expressions are comparable between Eastern and Western HCC cases.

## 1. Introduction

Liver cancer is one of the most common cancers, being the second cause of cancer-related death worldwide in which hepatocellular carcinoma (HCC) accounts for about 90% of liver cancer cases [1,2]. The incidence of HCC is closely associated with its known underlying etiologies, mostly due to liver cirrhosis caused by chronic viral hepatitis B and C infection, alcohol abuse, and aflatoxin exposure [3]. In Eastern Asia and Africa, the highest factor is a chronic infection of hepatitis B virus (HBV), whereas, in Western countries and Japan, chronic infection of hepatitis C virus is the highest risk factor [4]. Thus, the incidence of HCC shows a significant geographical distribution, with the highest number of cases in Eastern Asia predominantly being HBV-related. 

HCC is a vast heterogeneous tumor, mostly caused by various etiological factors and oncogenic transformations, which lead to histological diversity, varied tumor progression, and distinct molecular signatures [5,6]. The search for a common biomarker that could represent hepatocarcinogenesis in diverse HCC cases will be useful not only in a basic study but also in the development of diagnostic tools or potential treatment.

In the cellular context, various cell types co-exist in HCC tissues with their specific functions. One of the most important cellular populations, the cancer stem cells (CSC), are the tumorigenic cells within the tumor mass with normal stem cell properties such as self-renewal and multi-differentiation into multiple cell types [7]. These cells are important not only due to their cancer-initiating capacity, but also because of their chemo- and radio-resistance behavior [8,9,10], and recently also their capacity to escape molecular targeting therapy [11,12]. 

Multiple CSC markers have been used to identify hepatic CSC, with the most common ones the CD90/Thy-1, CD133/Prom-1, and CD326/EpCAM. The combination of these markers determines additional subpopulations in one CSC population, resulting in a wide variety of hepatic CSC phenotypes, also within a single tumor [13]. In the clinical setting, high expressions of CSC markers CD90, CD133, and EpCAM in HCC tissues had been associated with cellular differentiation, poor prognosis, and metastasis [14,15,16,17,18]. 

Currently, available data was mostly based on a specific patient population, mostly in Asian studies. Since HCC is multi-factorial and geographically-specific, in this study we demonstrated that the mRNA expression patterns of CSC markers, especially CD90, can be used to study both Western and Eastern populations, showing their relevance in hepatocarcinogenesis.

## 2. Materials and Methods

### 2.1. Human Liver Tissues

We analyzed two distant populations of selected HCC patients undergoing partial hepatectomy, 52 patients from Vietnam, and 43 patients in Italy. From each patient, three different portions of liver tissue were collected, consisting of HCC, peri-HCC, and distant non-tumoral tissue (distal) for a total of more than 200 tissue samples. Demographic data were recorded together with clinical findings including histology, laboratory measurements, tumor parameters, and treatment outcomes. Informed consent to participate in the study was obtained from each patient or by a legal representative. The study was approved by the ethical committees of the UMP Ho Chi Minh City no. 240/DHYD-HDDD and the Comitato Etico Regionale Unico of the Friuli Venezia Giulia, Prot. No. 18854. 

The protocol of sample collection (size, type of tissues, and storage condition) was agreed upon and standardized in both laboratories. Immediately after surgery, fresh liver tissues were collected and stabilized in RNAlater stabilizing solution (Invitrogen, Carlsbad, CA, USA) or snap-frozen in liquid nitrogen and stored at −80 °C. In parallel, liver tissues were fixed in formalin and included in the paraffin block. The fixed slices were subjected to Hematoxylin and Eosin (HE) and immunostaining. Two independent pathologists (UMC Ho Chi Minh city and ASUGI Trieste) performed histological analysis. The final diagnosis of patients was established in agreement based on international criteria together with its clinical findings. 

### 2.2. Total RNA Isolation and Reverse Transcription 

Protocol RNA extraction was standardized in both laboratories. Total RNA was extracted using the TriReagent (Sigma–Aldrich, St Louis, MO, USA) according to the manufacture’s protocol. RNA was quantified at 260 nm in a DU^®^730 spectrophotometer (Beckman Coulter, Fullerton, CA, USA). The integrity of RNA was assessed using an RNA 6000 Nano Kit in BioAnalyzer (Illumina, Agilent Technologies, St Clara, CA, USA) or gel electrophoresis and measuring the ratio A260/A280 with appropriate purity values between 1.8 and 2.0. 

### 2.3. Reverse Transcription—Quantitative Real-Time PCR (RT–qPCR)

RT-qPCR of all samples was performed in a single laboratory using identical controls, primer sets, and operators. Reverse Transcription (RT) was performed to obtain cDNA from 1 µg of purified RNA with the High Capacity cDNA Reverse Transcription Kits (Applied Biosystem, Foster City, CA, USA) according to the manufacture’s protocol. Real-time PCR was performed according to the SYBR Green Supermix protocol (Bio-Rad Laboratories, Hercules, CA, USA). PCR amplification was carried out in 15 µL reaction volume containing 25 ng cDNA, 1 × iQ5 SYBR Green Supermix—composed by 100 nM KCl, 40 nM Tris-Hcl, pH 8.4, 0.4 mM each dNTP, 40 U/mL iTaq DNA polymerase, 6 mM MgCl2, SYBR Green I, 20 mM fluorescein, and stabilizers—(Bio-Rad), and 250 nM of gene-specific forward and reverse primers. 

The primer sequences are designed using Beacon Designer 7.9 Software (PREMIER Biosoft International, Palo Alto, CA, USA) as follows: 18s-rRNA (5′-TAACCCGTTGAACCCCATT-3′ and 5′-CCATCCAATCGGTAGTAGCG-3′), ACTB (5′-CGCCGCCAGCTCACCATG-3′ and 5′-CACGATGGAGGGGAAGACGG-3′), CD90 (5′-AGAGACTTGGATGAGGAG-3′ and 5′-CTGAGAATGCTGGAGATG-3′), CD133 (5′-CATCTGCTCTCTGCTGAC-3′ and 5′-AACTTAATCCAACTCCAACC-3′) and EpCAM (5′-GAATAATAATCGTCAATGCCAGTG-3′ and 5′-CGCTCTCATCGCAGTCAG-3′). The reaction was run in the CFX 9600 or IQ5 real-time PCR system (Bio-Rad). Calculation of gene expression was based on cycling determination and was analyzed using the modification of the ΔΔCt equation, taking account on the efficiency of the reaction. Melting curve analysis was carried out to assess templates specificity.

### 2.4. Immunofluorescence 

The immunofluorescence (IF) analysis was performed on the paraffinated tissues section used for histological analysis. The tissue of the paired tumor and non-tumoral control was subjected to CD90 staining. After de-paraffinization with xylene and rehydration with a gradual concentration of ethanol, antigen retrieval was performed by microwave heat in 10 mM sodium citrate pH 6.0. The anti-human CD90-FITC conjugated antibody (Stem Cells Technologies, Vancouver, BC, Canada) was added and the nucleus was stained using Hoechst 33342. CD90 protein positivity was observed by using a fluorescence microscope Leica DM2000 (Leica Camera AG, Solms, Germany). 

### 2.5. Statistical Analysis

Continuous variables of CSC mRNA distribution as detected by RT-qPCR were given as a median (quartile 1—quartile 3; Q1—Q3). Graphics, survival curves, and statistical analyses were constructed using software GraphPad Prism version 5.01 (GraphPad Software, Inc., La Jolla, CA, USA). Differences within a cohort were calculated by non-parametric analysis with the Mann–Whitney test (two groups) and Kruskal–Wallis test with Bonferroni correction (three groups). For the comparison between two cohorts, a Chi-square analysis was performed. Survival was analyzed using Kaplan–Meier curves and the log-rank test. The cut-off value for the area under the curve (AUC) was defined by a receiver operating characteristic (ROC) curve plotting the true positive rate (sensitivity) against false positive rate (100—specificity). Statistical significance was set to 0.05 and presented as * *p* < 0.05, ** *p* < 0.01, *** *p* < 0.001.

## 3. Results

### 3.1. Patients’ Demographic and Clinical Baseline

The demographic and clinical features of the HCC patients in both cohorts are shown in Table 1. Both groups were mainly male for around 77% and 83% of Italian (Western) and Vietnamese (Eastern) patients, respectively. In the Eastern cohort, the mean age was significantly younger (*p* < 0.001), and as for HCC etiology, as expected, it was majorly related to HBV infection as compared to HCV/metabolic in Italian patients (*p* < 0.0001).

Regarding the HCC class and tumor parameter, no significant differences were noted between the cohorts. Most of the patients were Child-Pugh-Turcotte class A (88% and 93% for Italian and Vietnamese HCC, respectively). Also, for tumor nodule characteristics, most of the tumors were single nodules with no vascular invasion. Regarding the Edmonson-Steiner histological differentiation grade, both groups were predominantly well- and moderately-differentiated ES1-2 compared to poorly-differentiated ES3-4 for 77% vs. 23% for Italian and 71% vs. 29% for Vietnamese HCCs. 

For serum biochemical analysis, except for a significantly higher aspartate transaminase (AST) level in the Vietnamese group, no other differences were noticed for the levels of alanine transaminase (ALT) and alpha-fetoprotein (AFP). Mentioned as a median (Q1–Q3), the level of AST and ALT were 28 (23–73) and 28 (19–62) U/mL for Italian samples and 39 (29–75) and 38 (21–69) U/mL for Vietnamese samples. As for the AFP level, 56% Italian patients had an AFP level lower than 20 ng/mL, 17% between 20 ng/mL and 400 ng/mL, and 16% with AFP level above 400 ng/mL, while 31% Vietnamese patients had an AFP level lower than 20 ng/mL, 31% between 20 ng/mL and 400 ng/mL, and 21%with an AFP level above 400 ng/mL.

### 3.2. High Expression of CD90 mRNA Expression in the HCC Nodule

The relative mRNA expression in human tissue specimens was analyzed by RT-qPCR and expressed in arbitrary units (au) with a control sample considered as 1 au. By RTqPCR, we screened the expressions of the most common CSC markers: CD90 (Thy-1), CD133 (Prom-1), and EpCAM (CD326). The specificity and sensitivity of primer sets were tested on HCC cell lines expressing these markers (data not shown). 

**Figure 1:**
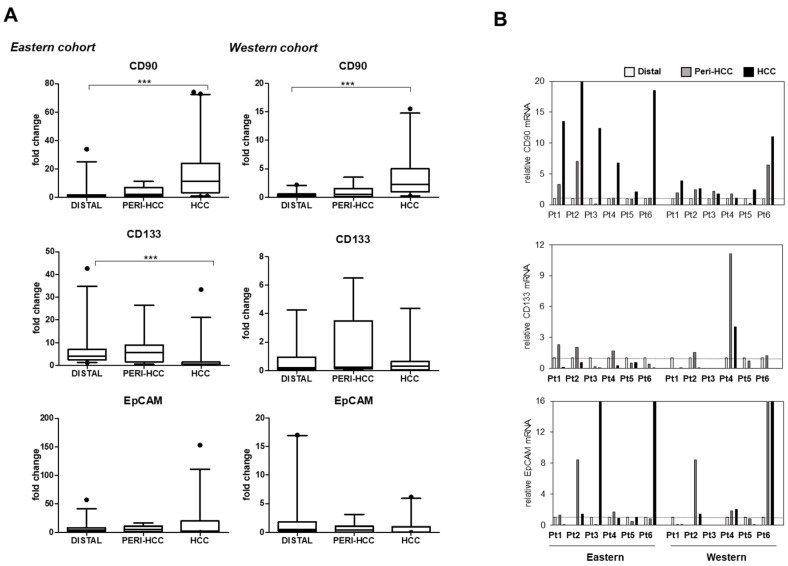
Distribution of CSC markers in the Eastern and Western cohorts. (**A**) mRNA expression of CSC markers CD90/THY-1, CD133/Prom-1, and EpCAM/CD326 in distal, peri-HCC, and HCC tissues. The expression of a sample was considered as 1.0. *** *p* < 0.001. Graphs are represented as median (bar) and 95% CI. (**B**) mRNA expression of CSC markers in six representative patients of each cohort shows clear up-regulation of CD90. Distal = 1.0, Pt = patient. The target gene was normalized to two reference genes ACTB and 18sRNA.

As shown in Figure 1A, comparable trends of CSC markers distribution between Italian and Vietnamese samples were noticed, in particular for CD90. The CD90 mRNA expression was progressively up-regulated from distal, peri-HCC, and highest in HCC nodules in both Vietnamese and Italian samples (*p* < 0.001 for HCC compared to peri-HCC and distal). The values were 0.99 (0.47–1.87) and 0.34 (0.13–0.57) for distal, 2.00 (0.92–6.87) and 0.49 (0.14–1.56) for peri-HCC, and 11.3 (3.30–24.03) and 2.25 (0.96–4.99) for HCC, for Vietnamese and Italian cohorts, respectively, represented as the median (Q1–Q3). The average extent of the up-regulation of CD90 in HCC compared to distal tissue was comparable between the two cohorts. 

In contrast to CD90, the CD133 mRNA in Vietnamese samples was down-regulated in HCC nodules compared to peri-HCC and distal portions (*p* < 0.001), while its distribution in Italian samples was highly variable. EpCAM mRNA expression was observed to be inconsistent in both groups and did not show any statistically significant values. The CD90 mRNA was expressed in all HCC nodules analyzed. On the other hand, the CD133 and EpCAM were absent in around 33% and 13% of samples, respectively. 

Figure 1B showed the expression of CSC markers in six representative patients from each cohort. The CD90 mRNA expression was up-regulated from distal, peri-HCC, to HCC, compared to variable expressions of CD133 and EpCAM.

### 3.3. CD90 mRNA Expression Is Related to HBV Infection 

Since the CD90 is a marker for several cell types in the liver, we then performed further analysis on the significance of CD90 mRNA expression in HCC nodules with clinical and pathological parameters. As shown in Table 2, CD90 expression was related to HBV infection (in the Vietnamese and combined cohort, *p* < 0.05) and poor-differentiation grade ES3-4 (in Italian cohort, *p* < 0.05). Further, a high CD90 mRNA level was significantly noticed in small tumors less than 5 cm in diameter (*p* < 0.05). 

### 3.4. HCC CD90 mRNA for Diagnostic and Prognostic Value

**Figure 2:**
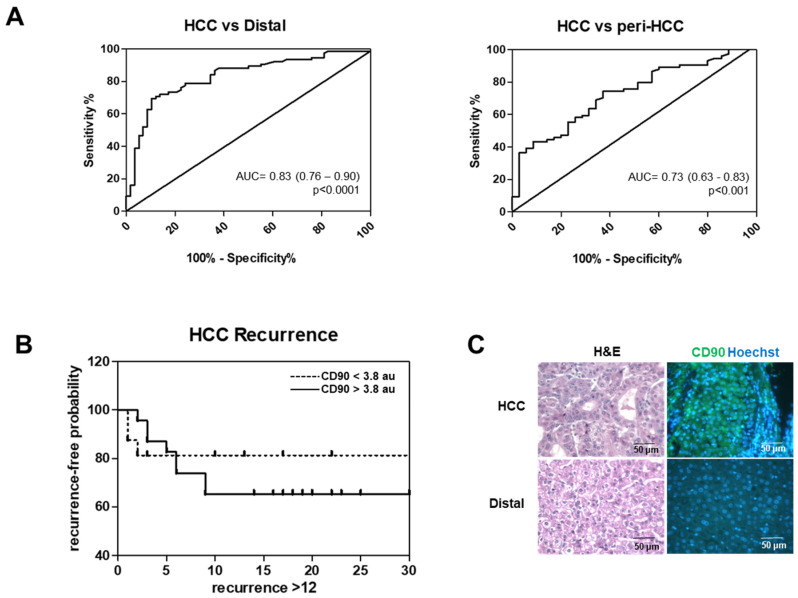
HCC CD90 mRNA expression for diagnostic and prognostic value. (**A**) Receiver operator characteristic (ROC) curve to distinguish HCC from distal (left) and peri-HCC (right) from Vietnamese and Italian cohorts. AUC = area under the curve. (**B**) Kaplan–Meier analysis of HCC recurrence in 12-months after hepatectomy. The graph represents 30-months of patient follow-ups. CD90 mRNA expression higher than 3.8 au is associated with a shorter recurrence time compared to lower expression. (**C**) Hematoxylin and Eosin (H & E) staining and immunofluorescence of CD90 in a representative paired HCC and distal tissue. CD90 protein is stained in green and the nucleus in blue. Magnification: objective 40×.

When considering the diagnostic value of CD90 mRNA, the area under the curve (AUC, 95% CI) of the receiver operator characteristic (ROC) curve was 0.83 (0.76–0.90) (*p* < 0.0001), distinguishing non-tumoral distal to HCC from all samples in both cohorts. Comparing HCC to peri-HCC, the AUC was slightly decreased to 0.73 (0.63–0.83) (*p* < 0.001) (Figure 2A). By using an ROC curve of HCC vs distal, a cut-off value of mRNA expression of 3.8 au was defined with 91% specificity.

Kaplan-Meier analysis was performed to associate the level of CD90 mRNA with HCC recurrence within one-year post-surgery in both cohorts (Figure 2B). We found that patients with HCC CD90 mRNA higher than 3.8 au had a shorter time to tumor recurrence compared to patients with lower CD90 with tumor recurrence mean (95% CI) of 15.4 (11.8–19.0) vs. 25.2 (12.6–37.7) months after surgery, respectively (*p* < 0.05). For the Kaplan–Meier analysis, even though no significant value was noticed, the difference between the two groups was noticed at around six-months post-surgery. The analysis of a separated cohort with adjusted cut-off values showed a similar trend (data not shown). This data indicated that HCC CD90 mRNA might have a positive prognostic value.

### 3.5. Positive Staining of CD90 Protein in the HCC Nodule

To investigate if the increased CD90 gene up-regulation was associated with an increased amount of protein, the CD90 protein content was assessed by IF in the paired tissue of HCC and its non-tumoral tissue. As shown in a representative photo in Figure 2C, CD90 positive cells were markedly present in HCC compared to the distal region. This confirmed the RNA distribution data.

## 4. Discussion

Due to its different etiologies, various risk factors, and long-term disease development, HCC tissue is vastly heterogeneous. Further, HCC, as other malignant solid tumors, exhibits a complex genetic diversity and genomic instability, driving tumorigenesis [19]. Heterogeneity of HCC is not only represented by the macro tumoral tissue organization and cancer cell morphology [20,21] but also in extensive –omics analysis. Extensive global transcriptomics studies identified subgroups of HCC associated with clinical and genetic characteristics, including HBV infection, host genetics, and aberrant molecular pathways [6,22]. 

Here we performed a simple comparative study on the distribution of most common CSC markers mRNA in two distinct populations, the Vietnamese and the Italian HCCs, representing the Eastern and Western cohort. The nature of HCC in Asian versus Western countries was different in terms of epidemiology and human genetics. From the disease management and therapy development, they approached different existing guidelines and consensus statements, and in clinical outcomes in clinical trials [23]. Thus, this information would be informative, not only in basic knowledge of hepatocarcinogenesis but also in the management of the disease. 

From this study, we observed the CD90 as the most significant marker of hepatocarcinogenesis compared to CD133 and EpCAM. CD90 mRNA was detected in 100% of samples analyzed compared to 67% and 87% for CD133 and EpCAM, respectively. Its distribution was progressively increased from the most distant part of the tumor to the border of HCC and is highest in the HCC nodule. Further, CD90 mRNA expression seemed to be positively correlated with the disease outcome of the patients. Patients with low CD90 in HCC nodules had later recurrence of HCC compared to patients with high CD90. 

CD90 (Thy-1) is a 25–37 kDa glycosylphosphatidylinositol (GPI)-anchored glycoprotein expressed in many cell types such as T cells, thymocytes, neurons, endothelial cells, and fibroblasts. It plays a relevant activity in the regulation of cell–cell and cell–matrix interaction, apoptosis, adhesion, migration, cancer, and fibrosis [24]. Several functions of CD90 have been described in physiological and pathological processes, mostly in its CD90 interactions with ligands such as integrins αv/β3, αx/β2, syndecan-4, CD90 itself, and CD97 [25]. CD90 was extensively studied in various types of cancers, as a tumor suppressor or tumor promoter [26], highlighting the importance of this molecule in the progression of the disease. In HCC, CD90 had been proposed as an important marker of hepatic CSC in various studies [10,27,28,29,30].

We had reported the expression of CD90 mRNA in a smaller number of liver tissues of Vietnamese patients [31] and previously, in Italian patients [32]. Here, we expanded the sample size also by adding the peri-HCC portion to observe the progression of liver disease in an individual patient. To perform a direct comparison between two cohorts, we standardized tissue collection and performed the analysis of all samples in a single laboratory with the same protocol allowing a direct assessment between two populations. Our data confirmed the importance of CD90 in hepatocarcinogenesis, including its correlation to HBV infection, poor histological grade, and prognosis [14,17].

As for diagnostic and prognostic value, this study has a limitation due to the tissue materials obtained from hepatectomy. Further, a cut-off value for relative mRNA expression could be variable between laboratories, thus an exact value might be different. However, this study has its strength since the patients did not receive previous treatment, thus representing the real natural course of hepatocarcinogenesis, also in an individual patient. Previous studies showed the clinical significance of the presence of CD90+ circulating tumor cells and circulating CSC in HCC [27,28,30,33], indicating the expression of CD90 either in in situ or in circulation is related to disease outcome. It should be also noted that in prostate cancer patients, proteomic analysis of urine identified a CD90 variant. Inversely, CD90 was not detected in the urine of post-prostatectomy patients [34]. This evidence provides another non-invasive detection and analysis of CD90. 

In summary, this comparative study showed that CD90 mRNA expressions are comparable between Eastern and Western HCC cases, suggesting a common indicator of hepatocarcinogenesis. A high level of CD90 mRNA can be associated with tumor recurrence, thus adding current knowledge for the development of future diagnostic and therapeutic tools. 

## Figures and Tables

**Table 1:** Demography and clinical-pathological characteristics of the HCC patients in the study.

Parameter	Eastern Cohort	Western Cohort	*p*
*n*	52	43	
Age (mean, 95%CI)	58 (55–61)	66 (63–69)	<0.001
Sex (M/F)	43/9	33/10	ns
Etiology			<0.0001
HBV (%)	33 (63%)	7 (16%)	
HCV (%)	9 (17%)	14 (33%)	
Metabolic/Alcohol (%)	10 (19%)	18 (42%)	
Others	0 (0%)	4 (9%)	
Disease scores			ns
CTP score A/B/C	43/3/0	38/5/0	
Tumor parameters			
Number (single/multiple/NA)	36/8/8	37/6	ns
Vascular invasion (yes/no/NA)	10/42/0	10/28/5	ns
ES grade (ES1/ES2/ES3-4/NA)	17/15/13/7	11/19/9/4	ns
Serum transaminases			
ALT (U/mL) [median (Q1–Q3)]	38 (29–75)	26 (19–62)	ns
AST (U/mL) [median (Q1–Q3)]	39 (29–75)	28 (23–73)	<0.05
Alpha fetoprotein			ns
<20 ng/mL	16	24	
20–400 ng/mL	16	8	
>400 ng/mL	11	7	
NA	9	4	

**Table 2:** Correlation between CD90 mRNA expression in HCC nodule and demography and clinical parameters of patients.

		Relative CD90 mRNA Expression in HCC (Median, Q1–Q3)		
Parameter		Eastern Cohort	Western Cohort	Both
Age	<65	10.5 (2.6–23.8)	1.0 (0.9–2.6)	7.3 (1.4–17.8)
	≥65	12.5 (4.5–33.7)	2.5 (0.4–5.4)	4.7 (1.6–13.1)
Sex	M	11.7 (4.1–25.7)	1.5 (0.5–6.5)	7.5 (1.3–16.3)
	F	9.4 (2.4–21.9)	2.5 (1.0–3.6)	3.0 (2.2–11.5)
Etiology	HBV	14.3 (6.3–34.5) *	1.0 (0.1–8.0)	12.9 (2.5–26.3) *
	HCV	12.1 (1.9–20.8)	2.6 (1.1–5.6)	2.8 (1.5–13.6)
	Metabolic	6.3 (2.5–11.5)	3.8 (0.6–8.6)	4.5 (2.3–11.5)
ES grade	ES 1–2	12.9 (2.7–26.3)	1.0 (0.4–2.6) *	3.1 (1.0–15.3)
	ES 3–4	8.6 (2.4–18.3)	3.7 (1.4–13.3)	6.9 (1.7–13.4)
AFP (ng/mL)	<20 ng/mL	12.1 (7.7–23.6)	2.2 (1.0–5.6)	5.8 (1.6–13.8)
	>20 ng/mL	11.3 (2.2–29.6)	1.4 (0.3–4.5)	4.8 (1.3–17.2)
HCC diameter	<5 cm	16.8 (10.8–46.8) *	2.8 (1.2–7.2) *	8.6 (2.3–16.8) *
	>5 cm	11.2 (4.9–19.8)	0.5 (0.2–2.9)	4.3 (0.6–13.5)

Differences within a cohort were calculated by non-parametric analysis with the Mann–Whitney test (two groups) and Kruskal–Wallis test (three groups). For the comparison between two cohorts, a Chi-square analysis was performed. * *p* < 0.05.
